# Effects of Arginine Supplementation on Amino Acid Profiles in Blood and Tissues in Fed and Overnight-Fasted Rats

**DOI:** 10.3390/nu8040206

**Published:** 2016-04-08

**Authors:** Milan Holecek, Ludek Sispera

**Affiliations:** Department of Physiology, Charles University in Prague, Faculty of Medicine in Hradec Kralove, Hradec Kralove 500 38, Czech Republic; sisperal@seznam.cz

**Keywords:** nutritional supplements, arginine, amino acids, starvation, nutrition

## Abstract

Chronic arginine intake is believed to have favorable effects on the body. However, it might be hypothesized that excessive consumption of an individual amino acid exerts adverse effects on distribution and metabolism of other amino acids. We evaluated the effect of chronic intake of arginine on amino acid concentrations in blood plasma, liver, kidneys, and soleus and extensor digitorum longus muscles. Rats were fed a standard diet or a high-arginine diet (HAD) for two months. Half of the animals in each group were sacrificed in the fed state, and the other half after fasting overnight. HAD increased blood plasma concentrations of urea, creatinine, arginine, and ornithine and decreased most other amino acids. Arginine and ornithine also increased in muscles and kidneys; an increase of lysine was observed in both muscle types. Methionine, phenylalanine, threonine, asparagine, glycine, serine, and taurine decreased in most tissues of HAD fed animals. Most of the effects of HAD disappeared after overnight fasting. It is concluded that (i) enhanced dietary arginine intake alters distribution of almost all amino acids; and (ii) to attain a better assessment of the effects of various nutritional interventions, an appropriate number of biochemical measurements must be performed in both postprandial and postabsorptive states.

## 1. Introduction

**Figure 1:**
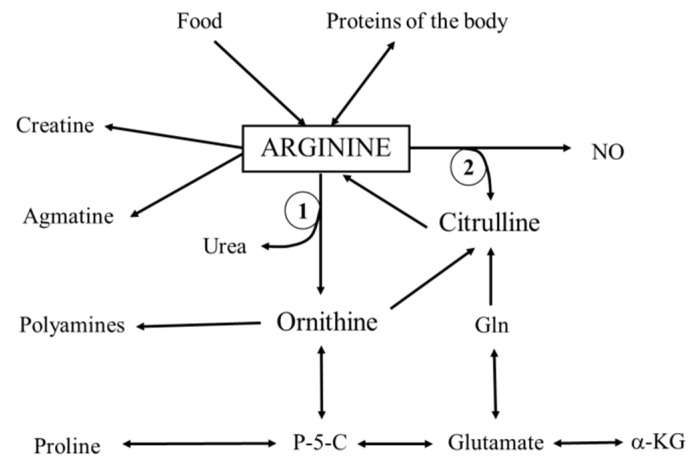
Overview of the main pathways of arginine metabolism. 1, arginase; 2, nitric oxide synthase; P-5-C, pyroline-5-carboxylate; α-KG, alpha ketoglutarate.

l-Arginine is a basic amino acid that is required for synthesis of proteins and serves as a precursor for synthesis of creatine, agmatine, urea, polyamines, proline, glutamate, and nitric oxide (Figure 1). l-arginine is classified as a conditionally essential amino acid because its endogenous synthesis may not be sufficient to meet metabolic demands in preterm infants and some cases of critical illness [1].

Current interest in l-arginine is focused mainly on its role in biosynthesis of nitric oxide and its stimulatory role in the secretion of insulin and growth hormone. Considerable literature exists from human and animal studies attesting to the fact that l-arginine may lower blood pressure, reduce blood clots and strokes, lower cholesterol and triglycerides, and improve diabetes and sexual functions via its role as a precursor for endothelium-derived nitric oxide [1,2,3]. There is no standard dose of arginine. A common dosage is 2 to 3 g three times a day; lower and higher doses have also been reported [4]. Of the available human studies, doses up to 20 g/day have been generally well tolerated. Minimal side effects such as nausea or diarrhea have been reported at higher doses [4,5].

Despite the many human and animal studies on arginine efficacy, there have been few studies investigating the specific effects of arginine supplementation on the distribution of amino acids in body fluids that may primarily result from alterations in arginine metabolism and amino acid transport across cell membranes. This may impair availability of some amino acids in a number of biochemical pathways and cellular functions, resulting in unexpected responses to various physiological and pathological conditions, such as starvation, exercise, trauma, infection, and cancer development.

The principle aim of the present study was to evaluate the effect of chronic intake of an arginine-supplemented diet on concentrations of free amino acids in selected tissues of white rats. The effect has been examined in two nutritionally different conditions—fed and overnight-fasted animals. In fed (postprandial) state, concentrations of nutrients in body fluids are closely related to the composition of the food and anabolic response of the body, mediated by enhanced secretion of insulin and activity of the parasympathetic system. After overnight fasting (postabsorptive state) food composition has a smaller effect on concentration of nutrients in extracellular fluid, and the main role it plays is the gradual decrease in insulin/glucagon ratio and enhanced catabolism of glycogen, lipids, and proteins. In this state, metabolic alterations are examined routinely in clinical practice.

## 2. Materials and Methods

### 2.1. Animals and Materials

Male Wistar rats (BioTest, Konarovice, Czech Republic) were housed in standardized cages in temperature-controlled quarters with a 12-h light-dark cycle. All rats received the standard laboratory diet (SLD) (Velas, Czech Republic) and drinking water *ad libitum*. The Animal Care and Use Committee of Charles University in Prague, Faculty of Medicine in Hradec Kralove specifically approved this study on 10 February 2010 (identification code: 24774/2006-11020). Chemicals were obtained from Sigma Chemical (St. Louis, MO, USA), Lachema (Brno, Czech Republic), Waters (Milford, MA, USA), Biomol (Hamburg, Germany), and Merck (Darmstadt, Germany).

### 2.2. Experimental Design

A total of 40 male Wistar rats at 7 weeks of age and weighing approximately 200 g each were randomly divided into two groups and fed an SLD or a high-arginine diet (HAD) for 2 months. HAD was prepared by mixing SLD (contains 24% of nitrogenous substances) with l-arginine (Sigma Chemical, St. Louis, MO, USA) in a ratio of 19:1. This resembles a high-dose supplementation of approximately 25 g of arginine per day in human subject.

At the end of the study the rats were sacrificed by exsanguination via the abdominal aorta and soleus (SOL) and extensor digitorum longus (EDL) muscles, liver, and kidneys were quickly removed and weighed. Half of the animals in each group were sacrificed in the fed state, the other half were sacrificed after an overnight fast. Small samples (of approximately 100 mg) of these tissues were immediately homogenized in 6% (v/v) perchloric acid, and the precipitated proteins were collected via centrifugation for 5 min at 12,000× *g*.

### 2.3. Amino Acid Concentrations in Blood Plasma and Tissues

Amino acid concentrations were identified in the supernatants of deproteinized blood plasma and tissue samples using high-performance liquid chromatography (Aliance 2695, Waters, Milford, MA, USA) after derivatization with 6-aminoquinolyl-*N*-hydroxysuccinimidyl carbamate. The intracellular concentration of each amino acid was calculated by subtracting the free extracellular portion from the total amount, assuming the plasma concentration to be equal to the concentration in the interstitial fluid as described by Bergstrőm *et al.* [6]. Total tissue water was measured from the tissue weight obtained after drying for 24 h at 90 °C. The determination of extra- and intracellular water was based on the chloride method according to Graham *et al.* [7].

### 2.4. Other Techniques

Plasma levels of urea, creatinine, ALT, AST, glucose, triglycerides, and cholesterol were measured using commercial tests (Boehringer, Mannheim, Germany; Elitech, Sées, France and Lachema, Brno, Czech Republic). Na^+^, K^+^, and Cl^−^ were determined with the help of ion-selective electrodes on AVL 983-S (Block Scientific, Englewood, NJ, USA).

### 2.5. Statistical Analyses

Results are expressed as means ± SE. Analysis of variance (ANOVA) followed by Bonferroni multiple comparison post hoc analysis was used to detect differences between multiple independent groups. NCSS 2001 statistical software (Kaysville, UT, USA) was used for analyses. Differences were considered significant at *p* < 0.05.

## 3. Results

### 3.1. Alterations in Food Intake, Body Weight Gain, and Weight and Protein Content of Tissues

We did not find significant differences in food intake, body weight gain, and weight and protein content of liver, EDL, and SOL between SLD and HAD fed animals. Significantly higher weight and protein content values were observed in the kidneys of animals fed by HAD. The effect was more pronounced in a fed state than after overnight starvation (Table 1 and Table 2).

### 3.2. Alterations in Blood Plasma

Standard blood biochemistry assays have shown that HAD increased concentrations of urea and creatinine and decreased potassium, triglycerides, and atherogenicity index (Table 3). Consumption of HAD increased blood plasma concentrations of arginine and ornithine and decreased a number of both essential (histidine, lysine, methionine, phenylalanine, threonine, and valine) and non-essential (asparagine, aspartate, glycine, proline, serine, taurine, and tyrosine) amino acids (Table 4). Plasma concentrations of isoleucine, leucine, alanine, glutamine, citrulline, and glutamate were unaffected. Most of the differences observed between SLD- and HAD-fed animals disappeared after overnight fasting. The exceptions were lower concentrations of citrulline, glutamine, glycine, and serine in animals fed before starvation by HAD than in animals fed by SLD.

### 3.3. Alterations in Tissues

Consumption of HAD increased arginine and ornithine and decreased intracellular concentration of a number of amino acids (particularly of methionine, phenylalanine, threonine, asparagine, glycine, serine, and taurine) in most of the examined tissues. The exception was the liver, in which arginine concentration was unchanged. A unique effect observed in skeletal muscle was an increase of lysine. After overnight fasting, the differences in amino acid concentrations between animals fed by SLD and HAD were mostly insignificant (Table 5, Table 6, Table 7 and Table 8).

## 4. Discussion

To the best of our knowledge, this is the first study assessing the specific effects of arginine supplementation on the distribution of amino acids in body fluids in postprandial and postabsorptive states. The data clearly demonstrate that chronically enhanced arginine intake leads to marked alterations in aminoacidemia in both the blood and tissues. As differences in food intake and weight gain between animals fed by SLD and HAD have been insignificant, the observed alterations are clearly due to the replacement of 5% of the SLD by l-arginine. The alterations are related to both the specific effects of arginine and effects of enhanced intake of nitrogen.

### 4.1. Alterations in Arginine Levels

As expected, chronic intake of HAD enhanced arginine concentrations in blood plasma, both types of skeletal muscle, and kidneys. The finding of unaffected arginine concentration in hepatic tissue may be explained by the response of the liver to enhanced arginine availability. Exogenous arginine induces arginase expression [8] and activates *N*-acetylglutamate synthase that catalyses production of *N*-acetylglutamate, which is an allosteric cofactor for carbamoyl phosphate synthetase I that acts as the controller of flux through urea cycle [9]. Therefore, unaltered concentrations of arginine in the liver are probably due to its activated catabolism as indicated by increased urea concentrations in blood plasma.

### 4.2. Alterations in Arginine Metabolites

It is believed that enhanced arginine availability and arginase activities have a role in synthesis of ornithine, glutamate, proline, and citrulline [10]. Markedly increased ornithine concentrations in plasma, muscles, and kidneys indicate that chronic consumption of arginine activates arginase not only in the liver, but also in extrahepatic tissues.

However, the effect of HAD on glutamate and citrulline was less pronounced, and even a decrease of proline was observed in blood plasma, liver, and kidneys. These findings indicate that other metabolic pathways or alterations in amino acid transport across plasma membranes have more important influences on glutamate and proline concentrations than enhanced availability of one of their precursors. Limited influence of HAD on citrulline levels is in agreement with the general opinion that the amount of citrulline produced via NO synthase is small, and that the main source of circulating citrulline is its synthesis from glutamine in small intestine.

### 4.3. Alterations in Other Amino Acids

One remarkable effect of arginine consumption was the decrease in concentration of a number of amino acids (particularly of methionine, phenylalanine, threonine, asparagine, glycine, serine, and taurine) in both blood plasma and tissues. The decrease of aminoacidemia in animals fed by HAD is markedly different from the minimal changes observed in animals chronically fed a high-protein diet [11]. A marked decrease (more than 10%) was found in EDL and kidneys. We believe this is a new and important finding, indicating a possible negative influence of abundant consumption of arginine. The lack of amino acids may exert a negative influence on various metabolic pathways, particularly on protein synthesis.

We suppose that the main cause of the decrease in aminoacidemia is the effect of a high concentration of arginine on the rate of transport of various amino acids across the cell membranes and not decreased availability of amino acids in the food. This suggestion is supported by unaltered concentrations of branched-chain amino acids, particularly leucine and isoleucine, which use different transporters than arginine [12].

Greater decreases in amino acid concentration in EDL (muscle composed of white, fast-twitch fibers) than in SOL (muscle composed of red, slow-twitch fibers) corresponds with our previous reports demonstrating that white muscles are more sensitive to various physiological or pathological signals than muscles containing mostly red fibers [13,14,15,16].

A noteworthy effect of chronic arginine consumption is an increased concentration of lysine in skeletal muscle. It has been shown that this tissue has a very high affinity for free lysine, and it has been suggested that muscle represents a major storage organ for free lysine [17]. Bergstrom *et al.* [18] have observed relatively greater elevations of lysine in muscle than of other indispensable amino acids after a protein-rich meal. However, the mechanism by which protein-rich meals or HAD increase lysine concentration in muscle is obscure.

### 4.4. Other Alterations Induced by HAD

Among the results of the effects of HAD obtained by standard blood biochemistry, the decrease of potassium in blood plasma is of special interest. This contradicts reports of its increase in acute studies in which arginine was infused as arginine monohydrochloride [19,20,21]. It has been suggested that the rise of potassium may be due to the exchange of cellular potassium for the proton from the arginine hydrochloride [20]. The decrease of potassium in our study, in which a pure l-arginine was used as a supplement, may be related to the well-known stimulatory effect of arginine on insulin secretion. The observed decrease in plasma triglycerides and atherogenicity index is in agreement with other studies reporting the triglyceride-lowering effect of l-arginine [22]. Higher weight and protein content of the kidneys in HAD fed animals is probably related to enhanced intake of nitrogen and not to the specific effect of arginine. We did not find an increase in weights of extensor digitorum longus and soleus muscles in animals fed by HAD as reported recently by Yang *et al.* [23].

### 4.5. Effect of Overnight Starvation

Most of the differences observed between animals fed by SLD and HAD in a fed state disappeared after overnight fasting. Minimal changes in amino acid concentrations have also been reported in animals fed a high-arginine diet that fasted 5 h before collection of blood samples for amino acid analysis [23]. These findings indicate that the body can maintain aminoacidemia in physiological ranges in conditions of excessive intake of arginine, and that laboratory biochemistry that is routinely performed after overnight fasting should also be performed in postprandial states in some conditions. It is noteworthy that overnight starvation did not decrease concentrations of arginine that were expected due to supposed up-regulation of arginine catabolizing enzymes, particularly arginase.

## 5. Conclusions

We conclude that enhanced dietary arginine intake has a significant effect upon the tissue distribution of all amino acids. The increase of arginine and ornithine and a decrease in concentration of a number of amino acids in blood plasma and tissues are among the main findings observed in animals sacrificed in postprandial state. The increase of lysine in skeletal muscle and the decrease of potassium, triglycerides, and atherogenicity index in blood plasma should also be noted. Observation that most of the alterations occurring in the fed state disappeared after overnight starvation indicates that some routine biochemical measurements should be performed in the postprandial state to get a clear picture of the effects of various nutritional interventions.

## Figures and Tables

**Table 1:** Changes in body weight and food intake in animals fed by SLD or HAD.

	SLD (*n* = 20)	HAD (*n* = 20)
Body weight (g)		
initial	198 ± 4	200 ± 5
1 week	257 ± 6	247 ± 6
6 weeks	450 ± 10	438 ± 10
70 days	525 ± 12	498 ± 12
Food intake (g/kg b.w./day)		
1st week	116 ± 4	122 ± 4
6th week	74 ± 1	73 ± 1
last week	64 ± 2	66 ± 1

Means ± SE. SLD, rats fed by standard laboratory diet; HAD, rats fed by arginine-enriched diet.

**Table 2:** Effect of chronic intake of HAD on tissue weights, protein content, and protein concentration in fed and overnight-starved animals.

	Fed Animals		Overnight-Starved Animals	
	SLD (*n* = 10)	HAD (*n* = 10)	SLD + S (*n* = 10)	HAD + S (*n* = 10)
Liver				
weight (g/kg b.w.)	32.42 ± 0.63	33.34 ± 1.16	23.13 ± 0.42	23.66 ± 0.44
protein (mg/g wet t.w.)	160 ± 9	147 ± 4	168 ± 4	156 ± 3
protein (g/kg b.w.)	5.17 ± 0.29	4.88 ± 0.18	3.88 ± 0.11	3.70 ± 0.10
Kidney				
weight (g/kg b.w.)	2.91 ± 0.06	3.45 ± 0.07 *	2.85 ± 0.09	3.22 ± 0.08 *
protein (mg/g wet t.w.)	128 ± 2	125 ± 4	126 ± 3	115 ± 3
protein (g/kg b.w.)	0.37 ± 0.01	0.43 ± 0.01 *	0.36 ± 0.01	0.37 ± 0.01 ^#^
EDL				
weight (g/kg b.w.)	0.43 ± 0.01	0.41 ± 0.01	0.43 ± 0.01	0.45 ± 0.01
protein (mg/g wet t.w.)	150 ± 3	146 ± 3	150 ± 3	145 ± 4
protein (g/kg b.w.)	0.06 ± 0.00	0.06 ± 0.00	0.06 ± 0.00	0.07 ± 0.00
SOL				
weight (g/kg b.w.)	0.46 ± 0.01	0.46 ± 0.01	0.48 ± 0.01	0.50 ± 0.01
protein (mg/g wet t.w.)	127 ± 4	128 ± 4	129 ± 3	118 ± 2
protein (g/kg b.w.)	0.06 ± 0.00	0.06 ± 0.00	0.06 ± 0.00	0.06 ± 0.00

Means ± SE, *p* < 0.05. * effect of arginine (HAD *vs.* SLD or HAD + S *vs.* SLD + S); ^#^ effect of starvation (SLD + S *vs.* SLD or HAD + S *vs.* HAD). SLD, rats fed by standard laboratory diet; HAD, rats fed by arginine-enriched diet.

**Table 3:** Effect of chronic intake of HAD on blood biochemistry in fed and overnight-starved animals.

	Fed Animals		Overnight-Starved Animals	
	SLD (*n* = 10)	HAD (*n* = 10)	SLD + S (*n* = 10)	HAD + S (*n* = 10)
Glucose (mmol/L)	10.7 ± 0.2	10.2 ± 0.2	9.1 ± 0.3 ^#^	9.9 ± 0.1 *
Urea (mmol/L)	7.1 ± 0.2	10.4 ± 0.4 *	6.6 ± 0.2	7.1 ± 0.2 ^#^
Creatinine (µmol/L)	27.6 ± 0.8	33.1 ± 0.9 *	31.8 ± 1.5 ^#^	30.2 ± 1.1
Sodium (mmol/L)	142.3 ± 0.5	141.6 ± 0.4	143.1 ± 0.3	142.5 ± 0.2
Potassium (mmol/L)	4.4 ± 0.1	3.8 ± 0.1 *	3.8 ± 0.1 ^#^	3.8 ± 0.1
Chloride (mmol/L)	100.9 ± 0.6	102.1 ± 0.5	103.2 ± 0.3 ^#^	102.3 ± 0.5
ALT (µkat/L)	0.9 ± 0.0	0.9 ± 0.0	0.6 ± 0.1 ^#^	0.7 ± 0.0
AST (µkat/L)	1.3 ± 0.1	1.5 ± 0.1	1.2 ± 0.0	1.2 ± 0.0 ^#^
Cholesterol (mmol/L)	1.8 ± 0.1	1.8 ± 0.1	1.5 ± 0.1	1.4 ± 0.1
HDL cholesterol (mmol/L)	1.1 ± 0.1	1.2 ± 0.1	1.1 ± 0.1	1.2 ± 0.0
LDL cholesterol (mmol/L)	0.3 ± 0.0	0.4 ± 0.0	0.3 ± 0.0	0.2 ± 0.0 ^#^
Atherogenicity index	0.7 ± 0.0	0.5 ± 0.0 *	0.3 ± 0.0 ^#^	0.3 ± 0.0 ^#^
Triglycerides (mmol/L)	1.6 ± 0.2	1.2 ± 0.1 *	1.0 ± 0.2 ^#^	0.8 ± 0.1 ^#^
Total protein (g/L)	63.7 ± 0.8	62.6 ± 0.5	61.1 ± 0.5 ^#^	62.5 ± 0.6
Albumin (g/L)	38.6 ± 0.9	40.1 ± 0.7	39.5 ± 0.5	40.4 ± 0.3

Means ± SE, *p* < 0.05. * Effect of arginine (HAD *vs.* SLD or HAD + S *vs.* SLD + S); ^#^ effect of starvation (SLD + S *vs.* SLD or HAD + S *vs.* HAD). Atherogenicity index was calculated as: (cholesterol—HDL cholesterol)/HDL cholesterol. SLD, rats fed by standard laboratory diet; HAD, rats fed by arginine-enriched diet.

**Table 4:** Effect of chronic intake of HAD on amino acid concentrations in blood plasma (µmol/L) in fed and overnight starved animals.

	Fed Animals		Overnight-Starved Animals	
Plasma	SLD (*n* = 10)	HAD (*n* = 10)	SLD + S (*n* = 10)	HAD + S (*n* = 10)
*Essential amino acids*				
Histidine	63 ± 1	54 ± 2 *	54 ± 2 ^#^	55 ± 1
Isoleucine	84 ± 2	81 ± 3	93 ± 2 ^#^	89 ± 3
Leucine	148 ± 5	141 ± 5	149 ± 6	152 ± 4
Lysine	293 ± 8	257 ± 7 *	308 ± 8	289 ± 10 ^#^
Methionine	53 ± 1	41 ± 1 *	48 ± 1 ^#^	48 ± 2 ^#^
Phenylalanine	66 ± 2	55 ± 2 *	65 ± 2	65 ± 1 ^#^
Threonine	248 ± 7	123 ± 5 *	234 ± 8	216 ± 7 ^#^
Valine	189 ± 5	166 ± 5 *	179 ± 5	182 ± 5
∑ EAA	1143 ± 21	917 ± 26 *	1130 ± 24	1097 ± 27
*Non-essential amino acids*				
Alanine	507 ± 13	495 ± 23	387 ± 16 ^#^	377 ± 16 ^#^
Asparagine	59 ± 2	45 ± 3 *	58 ± 1	55 ± 2 ^#^
Aspartate	28 ± 21	17 ± 1 *	15 ± 1 ^#^	12 ± 1 ^#^
Glutamine	671 ± 16	624 ± 16	620 ± 15	544 ± 13 *
Glycine	297 ± 7	161 ± 7 *	364 ± 13 ^#^	244 ± 7 *^,#^
Serine	252 ± 9	155 ± 5 *	230 ± 6	198 ± 5 *^,#^
Taurine	570 ± 40	181 ± 15 *	241 ± 15 ^#^	192 ± 8
Tyrosine	85 ± 2	57 ± 3 *	81 ± 3	64 ± 3
*Arginine and its metabolites*				
Arginine	173 ± 5	387 ± 27 *	151 ± 7	132 ± 5 ^#^
Citrulline	71 ± 3	73 ± 2	70 ± 2	54 ± 2 *^,#^
Glutamate	123 ± 8	112 ± 7	104 ± 5	96 ± 5
Ornithine	53 ± 3	132 ± 25 *	41 ± 1	30 ± 1 ^#^
Proline	229 ± 15	180 ± 15 *	133 ± 3 ^#^	117 ± 4 ^#^
∑ NEAA-Arg	2883 ± 78	2223 ± 62 *	2345 ± 39 ^#^	1984 ± 46 *^,#^
∑ AA-Arg	4026 ± 95	3150 ± 78 *	3475 ± 58 ^#^	3081 ± 69 *

Means ± SE, *p* < 0.05. * effect of arginine (HAD *vs.* SLD or HAD + S *vs.* SLD + S); ^#^ effect of starvation (SLD + S *vs.* SLD or HAD + S *vs.* HAD). SLD, rats fed by standard laboratory diet; HAD, rats fed by arginine-enriched diet.

**Table 5:** Effect of chronic intake of HAD on amino acid concentrations in liver (µmol/L of intracellular water) in fed and overnight-starved animals.

	Fed Animals		Overnight-Starved Animals	
Liver	SLD (*n* = 10)	HAD (*n* = 10)	SLD + S (*n* = 10)	HAD + S (*n* = 10)
*Essential amino acids*				
Histidine	1688 ± 41	1486 ± 29 *	1424 ± 43 ^#^	1390 ± 20
Isoleucine	379 ± 14	342 ± 19	386 ± 12	359 ± 16
Leucine	652 ± 42	754 ± 109	619 ± 21	646 ± 28
Lysine	853 ± 45	885 ± 50	1107 ± 40 ^#^	1132 ± 53 ^#^
Methionine	103 ± 5	92 ± 6	97 ± 8	83 ± 3
Phenylalanine	237 ± 9	214 ± 15	238 ± 10	227 ± 11
Threonine	894 ± 61	472 ± 23 *	970 ± 73	775 ± 47 *^,#^
Valine	614 ± 38	500 ± 27 *	561 ± 19	561 ± 26
∑ EAA	5420 ± 178	4746 ± 238 *	5401 ± 136	5173 ± 157
*Non-essential amino acids*				
Alanine	7860 ± 326	7786 ± 373	3871 ± 351 ^#^	5073 ± 329 ^#^
Asparagine	215 ± 8	180 ± 9 *	199 ± 11	174 ± 9
Aspartate	1740 ± 84	1477 ± 71	2014 ± 85	1827 ± 80 ^#^
Glutamine	13,485 ± 487	12,790 ± 474	13,693 ± 476	12,027 ± 365 *
Glycine	6602 ± 249	4270 ± 174 *	7977 ± 327 ^#^	6840 ± 158 *^,#^
Serine	1436 ± 126	656 ± 36 *	1170 ± 107	747 ± 59 *
Taurine	9388 ± 787	11,127 ± 865	10,420 ± 508	12,095 ± 896
Tyrosine	216 ± 12	185 ± 15	221 ± 13	186 ± 10
*Arginine and its metabolites*				
Arginine	48 ± 4	42 ± 6	20 ± 4 ^#^	46 ± 4 *
Citrulline	69 ± 4	78 ± 5	83 ± 6	71 ± 7
Glutamate	4234 ± 18	4893 ± 239 *	3879 ± 119	3599 ± 83 ^#^
Ornithine	810 ± 58	1010 ± 130 *	804 ± 33	638 ± 47 ^#^
Proline	387 ± 25	312 ± 14 *	325 ± 10	292 ± 21
∑ NEAA-Arg	46,441 ± 791	44,807 ± 1022	44,656 ± 818	43,568 ± 812
∑ AA-Arg	51,862 ± 909	49,511 ± 1184	50,058 ± 840	48,741 ± 859

Means ± SE, *p* < 0.05. * Effect of arginine (HAD *vs.* SLD or HAD + S *vs.* SLD + S); ^#^ effect of starvation (SLD + S *vs.* SLD or HAD + S *vs.* HAD). SLD, rats fed by standard laboratory diet; HAD, rats fed by arginine-enriched diet.

**Table 6:** Effect of chronic intake of HAD on amino acid concentrations in kidney (µmol/L of intracellular water) in fed and overnight-starved animals.

	Fed Animals		Overnight-Starved Animals	
Kidney	SLD (*n* = 10)	HAD (*n* = 10)	SLD + S (*n* = 10)	HAD + S (*n* = 10)
*Essential amino acids*				
Histidine	462 ± 22	349 ± 12 *	391 ± 15 ^#^	403 ± 20
Isoleucine	280 ± 14	254 ± 10	270 ± 13	261 ± 11
Leucine	529 ± 32	454 ± 20	493 ± 26	451 ± 17
Lysine	765 ± 31	636 ± 25 *	737 ± 37	730 ± 21
Methionine	110 ± 5	86 ± 5 *	102 ± 6	102 ± 6
Phenylalanine	232 ± 13	181 ± 8 *	209 ± 10	188 ± 7
Threonine	1395 ± 83	799 ± 30 *	1222 ± 56	1232 ± 37 ^#^
Valine	554 ± 28	453 ± 22 *	474 ± 25	481 ± 19
∑ EAA	4326 ± 200	3214 ± 114 *	3898 ± 157	3849 ± 119 ^#^
*Non-essential amino acids*				
Alanine	2198 ± 107	2019 ± 78	1653 ± 59 ^#^	1848 ± 46
Asparagine	381 ± 16	278 ± 14 *	331 ± 12 ^#^	313 ± 8
Aspartate	3819 ± 184	2644 ± 119 *	3184 ± 151 ^#^	3333 ± 128 ^#^
Glutamine	3609 ± 210	3370 ± 123	2932 ± 179 ^#^	2674 ± 102 ^#^
Glycine	7270 ± 217	4587 ± 170 *	7179 ± 384	6086 ± 193 *^,#^
Serine	2057 ± 83	1374 ± 38 *	1961 ± 83	2073 ± 92 ^#^
Taurine	33,328 ± 599	30,228 ± 1426	31,425 ± 658	32,065 ± 752
Tyrosine	367 ± 17	263 ± 14 *	314 ± 16	295 ± 21
*Arginine and its metabolites*				
Arginine	455 ± 15	663 ± 21 *	386 ± 19	430 ± 37 ^#^
Citrulline	61 ± 8	117 ± 7 *	55 ± 6	128 ± 8 *
Glutamate	16,320 ± 622	14,244 ± 712 *	13,971 ± 411 ^#^	14,388 ± 286
Ornithine	126 ± 19	214 ± 16 *	89 ± 6	107 ± 11 ^#^
Proline	546 ± 56	395 ± 20 *	350 ± 15 ^#^	346 ± 17
∑ NEAA-Arg	70,082 ± 1413	59,732 ± 1517 *	63,444 ± 1028 ^#^	63,656 ± 1226
∑ AA-Arg	74,408 ± 1588	62,946 ± 1578 *	67,342 ± 1144 ^#^	67,505 ± 1331

Means ± SE, *p* < 0.05. * effect of arginine (HAD *vs.* SLD or HAD + S *vs.* SLD + S); ^#^ effect of starvation (SLD + S *vs.* SLD or HAD + S *vs.* HAD). SLD, rats fed by standard laboratory diet; HAD, rats fed by arginine-enriched diet.

**Table 7:** Effect of chronic intake of HAD on amino acid concentrations in extensor digitorum longus muscle (µmol/L of intracellular water) in fed and overnight-starved animals.

	Fed Animals		Overnight-Starved Animals	
EDL	SLD (*n* = 10)	HAD (*n* = 10)	SLD + S (*n* = 10)	HAD + S (*n* = 10)
*Essential amino acids*				
Histidine	373 ± 17	391 ± 11	288 ± 11 ^#^	315 ± 9 ^#^
Isoleucine	134 ± 5	153 ± 4 *	162 ± 7 ^#^	174 ± 5 ^#^
Leucine	223 ± 6	223 ± 8	256 ± 13 ^#^	281 ± 7 ^#^
Lysine	782 ± 61	1034 ± 58 *	635 ± 35	657 ± 29 ^#^
Methionine	87 ± 3	63 ± 2 *	83 ± 4	86 ± 3 ^#^
Phenylalanine	123 ± 3	108 ± 4 *	122 ± 4	136 ± 3 *^,#^
Threonine	993 ± 31	602 ± 19 *	882 ± 34 ^#^	914 ± 15 ^#^
Valine	293 ± 10	290 ± 7	293 ± 13	335 ± 8 *^,#^
∑ EAA	3008 ± 109	2865 ± 84	2721 ± 102	2898 ± 50
*Non-essential amino acids*				
Alanine	3727 ± 143	4580 ± 118 *	3556 ± 158	3915 ± 112 ^#^
Asparagine	337 ± 18	279 ± 22 *	367 ± 13	402 ± 6 ^#^
Aspartate	524 ± 22	680 ± 20 *	754 ± 30 ^#^	960 ± 62 *^,#^
Glutamine	7538 ± 247	7362 ± 303	6424 ± 290 ^#^	5824 ± 221 ^#^
Glycine	4240 ± 300	2928 ± 99 *	4362 ± 346	4052 ± 246 ^#^
Serine	1497 ± 76	943 ± 31 *	1217 ± 37 ^#^	1168 ± 17 ^#^
Taurine	27,014 ± 467	21,951 ± 645 *	26,070 ± 597	24,280 ± 531 ^#^
Tyrosine	201 ± 6	150 ± 8 *	189 ± 7	175 ± 6 ^#^
*Arginine and its metabolites*				
Arginine	534 ± 34	2018 ± 195 *	393 ± 26	405 ± 17 ^#^
Citrulline	362 ± 12	416 ± 16 *	351 ± 14	297 ± 7 *^,#^
Glutamate	2536 ± 62	3066 ± 63 *	2493 ± 205	2757 ± 176
Ornithine	78 ± 6	314 ± 75 *	49 ± 3	44 ± 2 ^#^
Proline	569 ± 40	584 ± 43	343 ± 12 ^#^	381 ± 7 ^#^
∑ NEAA-Arg	48,623 ± 783	43,253 ± 840 *	46,175 ± 917	44,253 ± 654
∑ AA-Arg	51,631 ± 856	46,117 ± 904 *	48,896 ± 990	47,151 ± 687

Means ± SE, *p* < 0.05. * effect of arginine (HAD *vs.* SLD or HAD + S *vs.* SLD + S); ^#^ effect of starvation (SLD + S *vs.* SLD or HAD + S *vs.* HAD). SLD, rats fed by standard laboratory diet; HAD, rats fed by arginine-enriched diet.

**Table 8:** Effect of chronic intake of HAD on amino acid concentrations in soleus muscle (µmol/L of intracellular water) in fed and overnight-starved animals.

	Fed Animals		Overnight-Starved Animals	
SOL	SLD (*n* = 10)	HAD (*n* = 10)	SLD + S (*n* = 10)	HAD + S (*n* = 10)
*Essential amino acids*				
Histidine	759 ± 40	748 ± 28	655 ± 30	779 ± 43
Isoleucine	106 ± 5	120 ± 5	122 ± 4 ^#^	130 ± 4
Leucine	175 ± 8	172 ± 7	192 ± 6	209 ± 6 ^#^
Lysine	1327 ± 73	1704 ± 115 *	1562 ± 99	2012 ± 130 *
Methionine	74 ± 3	56 ± 2 *	68 ± 2	68 ± 2 ^#^
Phenylalanine	107 ± 4	92 ± 3 *	104 ± 2	113 ± 4 ^#^
Threonine	926 ± 19	607 ± 17 *	918 ± 36	1047 ± 34 *^,#^
Valine	228 ± 9	214 ± 8	222 ± 9	250 ± 5 ^#^
∑ EAA	3701 ± 108	3713 ± 136	3843 ± 143	4608 ± 179 *^,#^
*Non-essential amino acids*				
Alanine	3298 ± 129	3964 ± 167 *	3517 ± 215	3887 ± 214
Asparagine	636 ± 30	517 ± 36 *	743 ± 23	879 ± 42 *^,#^
Aspartate	2114 ± 213	2764 ± 285	3797 ± 193 ^#^	3351 ± 224
Glutamine	11,552 ± 464	11,408 ± 378	10,861 ± 403	11,224 ± 416
Glycine	2764 ± 88	2252 ± 50 *	3400 ± 92 ^#^	3293 ± 90 ^#^
Serine	2904 ± 127	1673 ± 56 *	2631 ± 104	2571 ± 108 ^#^
Taurine	33,561 ± 633	31,283 ± 467	32,960 ± 844	33,547 ± 659
Tyrosine	157 ± 5	111 ± 5 *	148 ± 6	132 ± 6 ^#^
*Arginine and its metabolites*				
Arginine	940 ± 53	3739 ± 186 *	871 ± 56	1006 ± 62 ^#^
Citrulline	586 ± 36	657 ± 22	646 ± 18	619 ± 34
Glutamate	5550 ± 121	6095 ± 137	6248 ± 221	6182 ± 286
Ornithine	128 ± 7	405 ± 69 *	95 ± 5	88 ± 4 ^#^
Proline	676 ± 49	724 ± 54	378 ± 9 ^#^	389 ± 12 ^#^
∑ NEAA-Arg	63,926 ± 1385	61,853 ± 1046	65,423 ± 1435	66,108 ± 1619
∑ AA-Arg	67,628 ± 1455	65,566 ± 1102	69,266 ± 1549	70,716 ± 1736

Means ± SE, *p* < 0.05. * effect of arginine (HAD *vs.* SLD or HAD + S *vs.* SLD + S); ^#^ effect of starvation (SLD + S *vs.* SLD or HAD + S *vs.* HAD).

## Abbreviations

The following abbreviations are used in this manuscript:

SLD: standard laboratory diet
HAD: high-arginine diet
EDL: extensor digitorum longus muscle
SOL: soleus muscle
EAA: essential amino acids
NEAA: non-essential amino acids
